# Normative data on the n-back task for children and young adolescents

**DOI:** 10.3389/fpsyg.2015.01544

**Published:** 2015-10-08

**Authors:** Santiago Pelegrina, M. Teresa Lechuga, Juan A. García-Madruga, M. Rosa Elosúa, Pedro Macizo, Manuel Carreiras, Luis J. Fuentes, M. Teresa Bajo

**Affiliations:** ^1^Department of Psychology, University of Jaén, Jaén, Spain; ^2^Department of Developmental and Educational Psychology, National University of Distance Education, Madrid, Spain; ^3^Department of Basic Psychology, National University of Distance Education, Madrid, Spain; ^4^Research Center for Mind, Brain and Behavior, University of Granada, Granada, Spain; ^5^Basque Center of Cognition, Brain and Language, San Sebastián-Donostia, Spain; ^6^Department of Basic Psychology and Methodology, University of Murcia, Murcia, Spain

**Keywords:** n-back, working memory updating, working memory development, memory development in children, working memory

## Abstract

The n-back task is a frequently used measure of working memory (WM) in cognitive neuroscience research contexts, and it has become widely adopted in other areas over the last decade. This study aimed to obtain normative data for the n-back task from a large sample of children and adolescents. To this end, a computerized verbal n-back task with three levels of WM load (1-back, 2-back, and 3-back) was administered to 3722 Spanish school children aged 7–13 years. Results showed an overall age-related increase in performance for the different levels of difficulty. This trend was less pronounced at 1-back than at 2-back when hits were considered. Gender differences were also observed, with girls outperforming boys although taking more time to respond. The theoretical implications of these results are discussed. Normative data stratified by age and gender for the three WM load levels are provided.

## Introduction

Working memory (WM) is a system that enables one to actively maintain and regulate a limited amount of task-relevant information (Baddeley and Logie, 1999). It plays an important role in processes like reading comprehension (e.g., García-Madruga et al., 1997; Cain, 2006), reasoning (e.g., Conway et al., 2003; García-Madruga et al., 2007), arithmetic calculations (e.g., Deschuyteneer et al., 2006), mathematical problem solving (e.g., Passolunghi and Pazzaglia, 2004), and academic performance (e.g., Alloway and Alloway, 2010). WM is also closely associated with fluid intelligence (e.g., Friedman et al., 2006).

WM has been traditionally measured using processing-and-storing dual tasks that involve performing a cognitive task while certain information has to be maintained in memory. For instance, in the Reading Span Test (Daneman and Carpenter, 1980), participants have to read increasing series of sentences aloud and eventually recall the last word of each sentence. In the counting span task (Case et al., 1982), subjects are presented with series of sets of figures, and they have to count the number of figures in order to recall this number at the end of the series. In the operation span task (Turner and Engle, 1989) participants are presented with a series of simple arithmetical equations and they have to verify each equation while memorizing a digit for later recall. In the analogy and anaphora span tasks (García-Madruga et al., 2005), participants have to resolve a series of analogies or anaphoric inferences and later recall the results of these inferences.

The n-back is a different kind of WM measure that does not involve a processing-and-storing dual task and in which individuals are not asked to recall any information but to recognize it. The n-back is a continuous recognition task in which participants must decide whether a stimulus was previously presented in certain conditions. In this task a sequence of items are shown, ranging from letters and drawings to words and numbers. For each item it must be determined whether the last one shown is identical to the stimulus presented “n” trials back. Therefore, at the 1-back level, each item needs to be compared with the one presented immediately before, that is, with the prior letter. At 2-back each stimulus is compared with the one shown two positions prior. At higher n levels the decision is based on stimuli separated each time by that many positions. Participants must respond “yes” or “no” to each trial. Response accuracy and response times are usually recorded.

First introduced by Kirchner (1958), the n-back is a widely used tool for assessing WM in the field of neuroscience. Its popularity in this area has much to do with how well it meets the demands of neuroimaging studies (Owen et al., 2005; Jaeggi et al., 2010). Thus, the responses that participants must provide are far simpler compared with other standard WM tasks; it is possible to record both accuracy and response times, and one can easily manipulate the level of difficulty depending on greater or lesser memory loads as well as the timing of stimulus presentation. Since the 1990s, as neuroimaging techniques have become more numerous, the n-back has grown in use and has been progressively adopted in other fields where other WM tasks were traditionally used.

The reliability of the task varies greatly depending on the study, from *r* = 0.16 (Van Leeuwen et al., 2007) to *r* = 0.91 (Friedman et al., 2008). The more complex levels such as 2-back and 3-back produce higher reliability coefficients (see Jaeggi et al., 2010). Correlations with other WM measures, although they differ from one study to another, are surprisingly low and not even significant (Redick and Lindsey, 2013). The variety of WM tasks and the different contents that they include (e.g., spatial, numeric, etc.) may be a reason for these low correlations. Thus, research findings where different types of WM measures (traditional or n-back) have been employed should not be considered as interchangeable (Kane et al., 2007).

Paradoxically, the n-back may show higher correlations with tasks that capture other constructs. In this regard, it is worth noting that relationships with intelligence measures are consistently found, in particular fluid intelligence, such as Raven’s Advanced Progressive Matrices (e.g., Kane et al., 2007; Colom et al., 2008; Jaeggi et al., 2010).

These seemingly contradictory findings may be related to the fact that different tasks capture different processes that contribute to WM performance. In this sense, the n-back includes components that have traditionally been considered parts of WM tasks. For example, a 2-back task requires participants to store two elements (e.g., letters) in WM. It further involves updating WM content, given that each time a new item is presented subjects must eliminate the previous item, add the new one, and maintain the presentation order of each item. In short, storage and processing operations that fall under the traditional definition of WM are also present in this task (Baddeley, 2007).

Furthermore, the n-back requires processes that are not found in other WM tasks. Perhaps the most relevant aspect is that n-back relies on recognition processes whereas traditional WM tasks require the retrieval of information. Hence, in n-back tasks participants may rely on assessments of familiarity, something that is not possible in traditional tasks. Moreover, n-back tasks depend on interference resolution processes in order to resolve the conflicts between familiarity and recollection, as well as other processes such as decision-making, temporal ordering, inhibition of no-longer-relevant information, and creation and updating of bindings between content and temporal context (see Oberauer, 2005; Kane et al., 2007).

The processes involved in the n-back task may also vary depending on the level of load. While all levels require active information maintenance, they differ not only in the load or quantity of information to maintain but also in the degree of content processing and involvement of executive control processes. Thus, at the 1-back level there is no need for content manipulation, whereas 2-back and above will involve constantly adding a new item and dropping the obsolete item. Moreover, content at 1-back is less susceptible to interference than at higher levels. At the 2-back level, participants have to store an element (e.g., a letter) for later use, and at the same time prevent it from influencing the response that they must provide. Thus control and interference resolution is crucial at higher load levels (Schleepen and Jonkman, 2010). On the other hand, it should be considered that the n-back is a recognition task, which may also involve familiarity and recollection. At 1-back, recognition could be based on familiarity. That is, because of its recency, the last presented item would likely have the higher level of activation in WM, which in turn would elicit a higher level of familiarity than other items in WM (see Oberauer, 2005). In contrast, at the 2-back (and higher) level, the decision may not only be based on familiarity given that different items could elicit stronger signals of familiarity than the item presented 2 or more steps back. Then a recollection process is needed in order to access the contextual information (e.g., order of presentation, temporal context) related to the item presented. Finally, the different levels vary according to the need to retrieve information outside the focus of attention in WM (Verhaeghen and Basak, 2005; Oberauer, 2006). Some models of WM (e.g., Cowan, 1995; Oberauer, 2002) differentiate between two embedded components: the focus of attention and an activated region of long-term memory. Oberauer (2002; see also McElree, 2001) assumed that the focus of attention is able to hold only one item that is directly accessible for processing operations. Thus, while at 1-back the necessary information to respond is in the focus of attention, at higher levels this information has to be accessed from outside of the focus. In short, 1-back and higher n-back levels have differing storage, processing, and executive control demands.

Working memory undergoes constant change through childhood and adolescence (e.g., Elosúa et al., 1997; Gathercole et al., 2004; Luna et al., 2004; Luciana et al., 2005), which in turn has an impact on the performance of different complex tasks (e.g., Siegel, 1994). In addition, developmental changes in the effectiveness with which information in WM is updated has been reported (e.g., Huizinga et al., 2006; Lechuga et al., 2006; Belachi et al., 2010; Lendínez et al., 2015). In recent years, some developmental studies using the n-back task have been conducted (Kwon et al., 2002; Vuontela et al., 2003; Schleepen and Jonkman, 2010). In general, this research has revealed gradual performance improvements during childhood (Vuontela et al., 2003). These changes continue through adolescence at the more demanding levels (i.e., 2-back; e.g., Schleepen and Jonkman, 2010).

The current study aims to provide normative data on n-back task performance among children and adolescents aged 7–13 years, contributing useful information on a task which is becoming increasingly more frequent and for which, to our knowledge, there is no normative data available. Previous studies have used only one or two levels of n-back in which the information had to be updated in memory. They have included only two or three groups of children of different ages for comparison with a rather limited number of children in each age group (e.g., less than 25). The present study used a more fine-grained approach to analyze the age trends in n-back performance through childhood and early adolescence. Thus, a wide range of ages, from 7 to 13 years, and a large sample for each of the ages was considered.

The current study also represents an opportunity to analyze age trends in the task. Given the nature of the process involved at the different levels of n-back, different age-related patterns for each n-back level might be obtained. Since the 2-back or 3-back versions require the involvement of higher executive control processes to a greater extent than the 1-back condition, we expected a more pronounced increase in performance with age in the more complex conditions than in the 1-back version of the task.

To this end, three levels of WM load (1-back, 2-back, and 3-back) were administered as part of a large-scale study assessing different cognitive functions considered essential for adequate academic performance, such as attention, memory, language, and reasoning.

## Materials and Methods

### Participants

A total of 3722 children and adolescents (1886 boys and 1836 girls) aged 7–13 years took part in this study. Only children whose parents consented to their participation in the study were administered the task. No other criteria were applied for exclusion. This was to obtain the largest sample size possible and to represent the full range of children as accurately as possible. The participants made up a sample of school children recruited from 43 schools across several Spanish cities. The number of participants in each age group, their gender and mean age is shown in Table 1. Prior to testing, informed consent to children’s participation was obtained from a parent or legal guardian. Approval from the ethical committee of the University of La Laguna (Spain) was obtained previous to the study.

**TABLE 1:** **Age groups of the study population**.

		**Gender**		**Age (months)**		**% Completed**		
**Age group**	***N***	**Boys**	**Girls**	***M***	***SD***	**1-back**	**2-back**	**3-back**
7	387	193	194	91.32	2.52	98.71	77.78	34.88
8	592	285	307	101.32	3.49	99.83	83.45	41.89
9	606	310	296	113.82	3.37	99.67	86.96	49.01
10	618	297	321	125.35	3.50	99.51	91.59	60.68
11	601	315	286	137.50	3.47	99.67	91.35	66.72
12	476	253	223	149.39	3.39	100.00	92.65	70.59
13	442	233	209	161.38	3.38	99.10	94.57	78.96
Total	3722	1886	1836					

Number, gender, and mean age of participants for each age group and percentage of participants completing each level of the task.

### Materials and Procedure

Children were given a series of cognitive tasks over three sessions as a part of a larger study on the development of cognitive skills involved in academic performance. The n-back task was administered at the beginning of the session. Each session lasted about 60 min.

The n-back task programmed by Robinson and Fuller (2004) in E-prime (Schneider et al., 2002) was used in this study. The version of the task applied here consisted of three levels: 1-back, 2-back, and 3-back. The items to be updated were letters, that is, linguistic material. The following 20 consonants were used as stimuli in all blocks: B, C, D, F, G, H, J, K, L, M, N, P, Q, R, S, T, V, W, Y, Z. Stimuli were presented one by one in the center of the screen (font: Palatino Linotype, size: 30). On the 1-back task, children had to compare the current trial to the previous one; they were asked to press the “yes” key only when the two subsequently presented letters were the same; otherwise they were to press the “no” key. The 2-back and 3-back trials were similar, with the exception that on 2-back trials, children had to compare the currently presented letter to the one presented two trials before, and on 3-back trials, to the letter presented three trials before. The first three trials of each block were always non-target trials where the correct response was “no.” Each stimulus appeared on the screen for 500 ms, followed by a screen that remained blank for another 3000 ms. The children had 3500 ms, from stimulus onset until the beginning of the subsequent trial to press the corresponding response key. Responses times as well as the number of hits, correct rejections, misses, false alarms, and non-responses were recorded.

Each level of the task (1-back, 2-back, etc.) was preceded by (1) instructions, (2) examples that included a sequence of six letters with the corresponding correct responses, and (3) a practice block that consisted of 20 trials (30% “yes” trials). And additional practice block was administered if a participant did not reach a correct percentage of 60% on the “yes” trials. There were two test blocks made up of 20 trials each (also with 30% “yes” trials), so that there were a total of 40 trials per level. In order to prevent fatigue and frustration, the task was discontinued when the children did not reach a correct percentage of 60% on a given test block.

### Data Analyses

Two sets of analyses were conducted. The first was carried out to determine the age trends across the different levels of the n-back task. To this end, separate analyses were performed on the percentage of correctly identified targets (hits), the percentage of false alarms (“yes” responses to non-targets), the signal-detection parameter d-prime (d′) that reflects the sensitivity of the participants to discriminate items as previously presented (or not) n trials back, and the mean response times for correctly identified targets.

The second set of analyses was aimed to provide normative data on the different levels of the n-back task and consisted of multiple regression analyses on accuracy that included the following predictors: age (in months), age squared, and gender.

## Results

### Age-Related Data

A mixed ANOVA was conducted at 1- and 2-back for each of the aforementioned variables, including age (7, 8, 9, 10, 11, 12, and 13 years) and gender as the between-group factors. If the more difficult level (3-back) had been included, the total sample would have been reduced by up to 42%. However, by keeping to the 1- and 2-back levels, 11% of participants were excluded. Table 1 shows the number of participants by age that performed levels 1 and 2 of the n-back task^1^.

#### Hits

A 2 (n-back load) × 7 (age) × 2 (gender) ANOVA was conducted on the percentage of hits. Table 2 shows the ANOVA summary table for all the dependent variables. There was a significant main effect of load, with the percentage of hits in the 1-back condition (*M* = 74.04, *SD* = 16.8) higher than that observed at 2-back (*M* = 50.55, *SD* = 25.22). The main effects were also significant for age and gender. The gender differences indicated that girls (*M* = 62.59, *SD* = 16.82) had a slightly higher percentage of hits than boys (*M* = 61.42, *SD* = 16.80). There was also a significant interaction between load and age. All other interactions failed to reach the standard significance level (*p* > 0.075).

**TABLE 2:** **Summary for ANOVAs performed on hits, false alarms, d-prime, and response time**.

	**Hits**				**False alarms**					**D-prime**			**Response times**			
**Source**	***F***	***df***	***P***	${\text{η}}_{p}^{2}$	***F***	***df***	***p***	${\text{η}}_{p}^{2}$	***F***	***df***	***p***	${\text{η}}_{p}^{2}$	***F***	***df***	***p***	${\text{η}}_{p}^{2}$
N-back load	3080.52	1,3282	0.000	0.48	554.69	1,3282	0.000	0.14	3703.15	1,3282	0.000	0.53	841.12	1,3107	0.000	0.21
Age	84.45	6,3282	0.000	0.13	23.92	6,3282	0.000	0.04	92.48	6,3282	0.000	0.14	84.52	6,3107	0.000	0.14
Gender	3.96	1,3282	0.047	0.01	8.75	1,3282	0.003	0.01	15.74	1,3282	0.000	0.01	57.46	1,3107	0.000	0.02
N-back load age	20.27	6,3282	0.000	0.04	1.06	6,3282	0.385	0.01	12.00	6,3282	0.000	0.02	0.69	6,3107	0.660	0.01
N-back load gender	1.24	1,3282	0.265	0.00	2.94	1,3282	0.086	0.01	6.61	1,3282	0.010	0.01	6.38	1,3107	0.012	0.01
N-back load age gender	0.41	6,3282	0.872	0.01	1.10	6,3282	0.362	0.01	0.66	6,3282	0.684	0.01	1.49	6,3107	0.176	0.01
Age gender	1.91	6,3282	0.075	0.01	0.32	6,3282	0.928	0.01	1.63	6,3282	0.134	0.01	1.56	6,3107	0.154	0.01
Age (linear)					475.76	1,3289	0.000	0.13					419.66		0.000	0.12
Age (linear) n-back load	108.16	1,3289	0.000	0.03		1,3289			50.92	1,3289	0.000	0.01				
Age (linear) in 1-back	208.90	1,3289	0.000	0.06		1,3289			273.75	1,3289	0.000	0.08				
Age (linear) in 2-back	415.71	1,3289	0.000	0.11		1,3289			502.58	1,3289	0.000	0.13				

**FIGURE 1:**
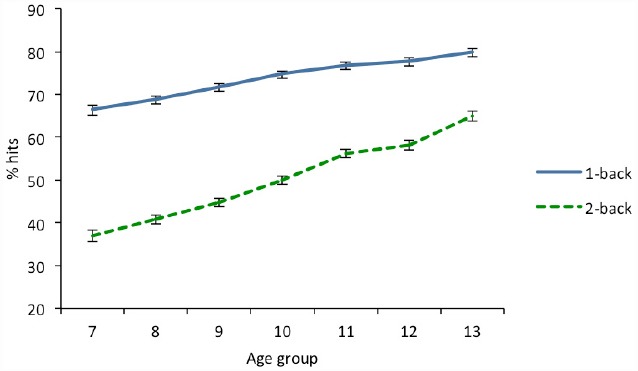
**Percentage of hits as a function of age in 1-back and 2-back conditions.** Error bars indicate two standard error of the mean.

There was an increase in the percentage of hits in relation to age, although the patterns observed for each n-back load were different (see Figure 1). This was confirmed by the crucial interaction between the linear trend for age and load. A trend analysis for each n-back load indicated a relatively small but significant linear increase in performance with age at the 1-back level and a more pronounced linear increase with age at 2-back.

A posteriori analyses using the Bonferroni method were conducted to determine between which age groups the differences occurred. At 1-back no differences were found between some of the contiguous age groups, that is, 7 vs. 8 (*p* = 1), 8 vs. 9 (*p* = 0.079), and 10 vs. 11 years (*p* = 0.762). From age 11, there were also no differences between the different groups (11, 12, and 13, *p* > 0.076). The remaining comparisons were significant (*p* < 0.001). At 2-back no differences were observed in some contiguous age groups, namely 7 vs. 8 (*p* = 0.487), 8 vs. 9 (*p* = 0.168), and 11 vs. 12 (*p* = 1). The rest of the comparisons between the different age groups showed statistically significant differences (*p* < 0.007).

#### False Alarms

**FIGURE 2:**
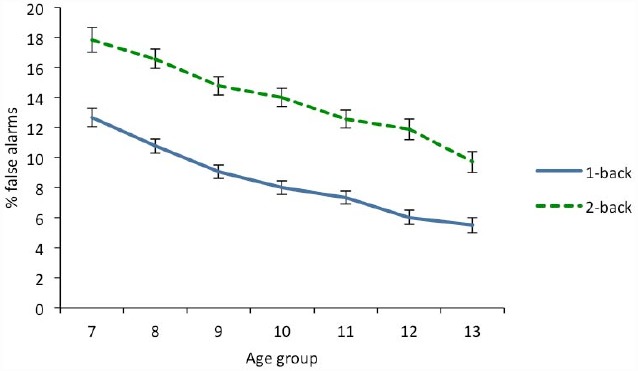
**Percentage of false alarms as a function of age in 1-back and 2-back conditions.** Error bars indicate two standard error of the mean.

A 2 (n-back load) × 7 (age) × 2 (gender) ANOVA was conducted on the percentage of false alarms (see Table 2). There was a significant effect of load, with the percentage of false alarms in the 1-back condition (*M* = 8.32, *SD* = 10.66) lower than that observed at 2-back (*M* = 13.81, *SD* = 14.56). The main effect of age was also significant. As can be seen in Figure 2, there was a linear decrease in the percentage of false alarms with age similar at both n-back levels. A trend analysis revealed that only the linear component was significant, accounting for 99.12% of the variance. A posteriori analyses using the Bonferroni correction revealed differences in recognition between each age group (*p* < 0.05), except at contiguous age groups (*p* > 0. 20) and between 10 and 12 year old (*p* = 0.06).

There were also a significant albeit small gender effect. This was caused by boys (*M* = 11.77, *SD* = 10.99) committing more false alarms than girls (*M* = 10.63, *SD* = 11.01). None of the interactions approached significance (*p* > 0.086).

#### D-Prime

D-prime was estimated as *d*′ = *Z_Hits_*–*Z_FalseAlarms_*. The fourth method proposed by Stanislaw and Todorov (1999) was used to avoid that *d*′ might be undetermined when the hit or the false-alarm rate was equal to 0 or 1. Specifically, scores equal to 0 were replaced by 0.5/*n* and scores equal to 1 were replaced by (*n* –0.5)/*n*, with *n* representing the number of signal and noise trials. A 2 (n-back load) × 7 (age) × 2 (gender) ANOVA was conducted on these values, similar to the previous analyses (see Table 2).

The ANOVA revealed a significant effect of load given that *d*′ was greater in the 1-back condition (*M* = 2.28, *SD* = 0.89) than at 2-back (*M* = 1.29, *SD* = 0.99). The main effects were also statistically significant for age and gender. In addition, the interactions of age by load and gender by load were also significant. All other interactions failed to reach the standard significance level (*p* = 0.134).

**FIGURE 3:**
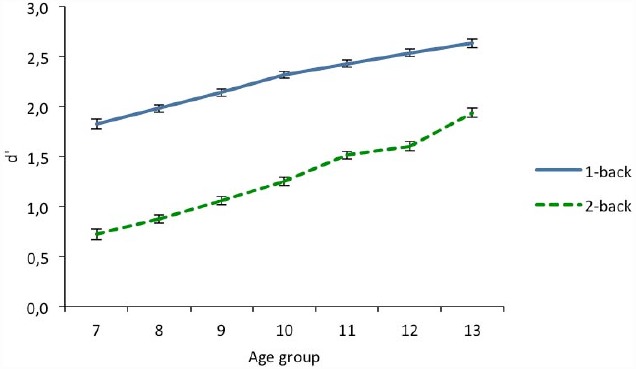
**D-prime scores (***d***′) as a function of age in 1-back and 2-back conditions.** Error bars indicate two standard error of the mean.

An interaction analysis between the linear trend of age and load revealed that even though *d*′ increased with age at both levels of load, the age trend was different for each level as shown by the significant interaction. As with the previous analysis on hits, an age-trend analysis for each n-back load indicated an age-related linear increase on *d*′ at both 1-back and 2-back. Thus, the interaction was again due to the fact that the linear increase was more pronounced at 2-back than at 1-back level (see Figure 3). This pattern was also evidenced by *post hoc* analyses with the Bonferroni correction that showed that at 1-back, all the age groups differed (*p* < 0.05), except at contiguous age groups (*p* > 0.06), excluding 9 vs. 10 (*p* = 0.009). At 2-back significant differences were observed between all age groups (*p* < 0.027), except between 7 vs. 8 (*p* = 0.514) and 11 vs. 12 (*p* = 1).

As regards the interaction between n-back levels and gender, a univariate analysis for each level revealed a significant gender difference on *d*′ at 1-back, *F*(1,3295) = 19.58, *p* < 0.001, given that girls (*M* = 2.34; *SD* = 0.85) showed a higher *d*′ than boys (*M* = 2.19, *SD* = 0.85). However, at 2-back, girls (*M* = 1.31, *SD* = 0.93) and boys (*M* = 1.25, *SD* = 0.93) obtained similar *d*′, *F*(1,3295) = 2.29, *p* = 0.13.

#### Response Times

Only response times for hits were analyzed. A 5% of participants were excluded from this analysis because they did not have hits at the 2-back level. These participants were not distributed evenly across all age groups: 97 children were excluded from the 7-year-old group, 21 form the 8-year-old group, 17 from the 9-year-old group, 14 from the 10-year-old group, 11 from the 11-year-old group, 7 from the 12-year-old group, and 8 from the 13-year-old group.

**FIGURE 4:**
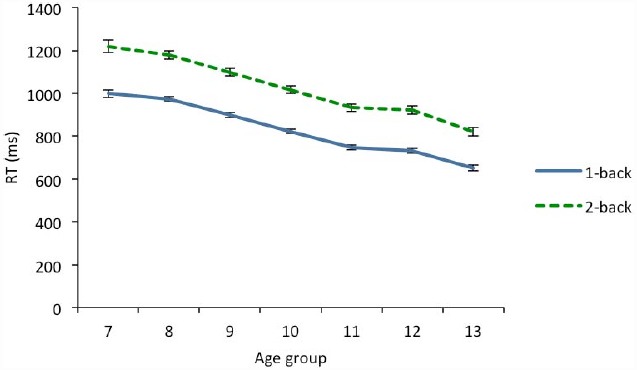
**Response time as a function of age in 1-back and 2-back conditions.** Error bars indicate two standard error of the mean.

The results of the mixed ANOVA revealed significant main effects for all variables (see Table 2). Load had a significant effect, since response times at 1-back were lower (*M* = 821.66; *SD* = 277.95) than at 2-back (*M* = 1014.32; *SD* = 432.22). An age effect was also found (see Figure 4). A trend analysis showed a significant linear component for age that explained 97.7% of the variance. Finally, there were a significant gender effect that was qualified by a significant interaction between load and gender. Although boys (*M* = 887.78, *SD* = 283.82) tended to respond quicker than girls (*M* = 970.94, *SD* = 284.44), this difference was greater at 2-back (106 ms) than at 1-back (73 ms) level. No other interaction was significant (*p* = 0.154). *Post hoc* comparison with the Bonferroni correction revealed significant differences between all age groups (*p* < 0.001), except between 11 and 12 years where no differences were found (*p* = 1).

Given that girls outperformed boys but took longer time to respond, there was the possibility of a speed-accuracy trade-off. To assess this explanation a complementary analysis of the inverse efficiency scores was performed (Townsend and Ashby, 1983). The inverse efficiency score is a composite index of response time and accuracy that takes speed-accuracy trade-offs into account. This score was calculated by dividing the mean correct response times by the proportion of hits for each participant and for each level of n-back. Then, a 2 (n-back load) × 7 (age) × 2 (gender) ANOVA was conducted on the inverse efficiency score. The expected main effects of load, *F*(1,3107) = 724.96, *p* < 0.001, $\eta_{p}^{2}$ = 0.19, and age, *F*(6,3107) = 51.58, *p* < 0.001, $\eta_{p}^{2}$ = 0.09, as well the interaction between load and age, *F* (6,3107) = 23.40, *p* < 0.001, $\eta_{p}^{2}$ = 0.043 were significant. More importantly, the main effect of gender reached significance, *F*(1,3107) = 6.90, *p* = 0.009, $\eta_{p}^{2}$ = 0.002, with girls showing a higher inverse efficiency score (2225) than boys (2030). Thus, the gender effect was again obtained with an index uncontaminated by possible speed-accuracy trade-offs.

### Normative Data

A set of multiple regression analyses on accuracy were carried out. The predictors were age (in months), age squared, and gender. By including age squared it was possible to assess the quadratic effects of age. Age was centered to avoid multicollinearity between predictors before calculating the quadratic term. Gender was coded as 1 for boys and 2 for girls.

The analyses of each level included all participants who carried out that level of the task. To analyze total hits, all participants were included and the hits for each level of load were summed. If participants had not performed the task beyond a certain level (e.g., 2-back), they were given a score equal to 0 on that and higher levels.

Regression models for the percentage of hits at each n-back level as well as for total hits are shown in Table 3. Results reveal that age was the strongest predictor, contributing to an increase in performance at all n-back levels as well as for total hits. The quadratic age component also predicted performance, but only at 1-back. This component showed that age-related increases in performance diminished with age. Gender was found to be a predictor only in the 1-back condition and for total hits. Girls obtained a higher number of hits than boys at both levels of the task.

**TABLE 3:** **Multiple linear regression models of percentage of total hits and percentage of hits on each version of the task with gender, age, and age squared as predictors**.

	**Variable**	***B***	***SE***	**Standardized*β***	***t***	***p***	***R*^2^**
Total (*n* = 3722)	(Constant)	19.05	0.50		37.94	0.000	
	Gender	0.93	0.30	0.05	3.09	0.002	
	Age	0.16	0.01	0.37	24.32	0.000	
	Age^2^	–0.01	0.00	–0.03	–1.68	0.092	0.14
1-back (*n* = 3722)	(Constant)	66.21	1.14		58.02	0.000	
	Gender	2.68	0.69	0.06	3.91	0.000	
	Age	0.27	0.01	0.28	17.52	0.000	
	Age^2^	–0.01	0.01	–0.05	–3.42	0.001	0.08
2-back (*n* = 3296)	(Constant)	49.09	1.38		35.59	0.000	
	Gender	0.83	0.82	0.02	1.01	0.317	
	Age	0.39	0.02	0.35	2.93	0.000	
	Age^2^	–0.01	0.01	–0.01	–0.61	0.541	0.12
3-back (*n* = 2141)	(Constant)	5.54	1.48		34.24	0.000	
	Gender	0.45	0.88	0.01	0.51	0.611	
	Age	0.23	0.02	0.24	1.73	0.000	
	Age^2^	0.00	0.01	–0.01	–0.20	0.844	0.06

Normative data stratified by age and gender for total hits and those obtained at each n-back level are shown in Table 4.

**TABLE 4:** **Normative data for total hits and for hits on each version of the task stratified by gender and age (7, 8, 9, 10, 11, 12, 13)**.

			**Boys (*n* = 1886)**							**Girls (*n* = 1836)**						
			**Age group**							**Age group**						
			**7**	**8**	**9**	**10**	**11**	**12**	**13**	**7**	**8**	**9**	**10**	**11**	**12**	**13**
			**(*n* = 193)**	**(*n* = 285)**	**(*n* = 310)**	**(*n* = 297)**	**(*n* = 315)**	**(*n* = 253)**	**(*n* = 233)**	**(*n* = 194)**	**(*n* = 307)**	**(*n* = 296)**	**(*n* = 321)**	**(*n* = 286)**	**(*n* = 223)**	**(*n* = 209)**
Total	*M*		14.45	16.71	17.63	20.23	21.94	22.5	24.82	14.25	15.58	18.70	21.07	23.24	24.70	27.20
	*SD*		8.49	9.20	9.17	9.44	9.90	9.66	9.85	8.58	8.41	9.29	9.10	9.28	9.21	8.74
	Percentile	5	3	3	4	4	4	4	4	2	3	4	5	5	7	11
		25	7	10	10	13	14	14	18	8	10	12	13	16	18	23
		50	13	15	16	21	24	24	28	13	15	18	22	25	26	30
		75	22	25	25	28	30	30	32	20	22	26	28	31	31	34
		95	28	32	32	35	35	37	37	30	30	34	35	35	38	39
1-back	*M*		8.05	8.82	9.14	9.72	10.01	10.20	10.28	8.21	8.60	9.54	10.11	10.41	10.73	11.29
	*SD*		3.04	3.09	2.82	2.96	2.98	2.96	2.96	3.29	3.09	3.12	2.86	2.77	2.55	2.34
	Percentile	5	3	3	4	4	4	4	4	2	3	4	5	5	6	7
		25	6	7	7	8	8	8	9	6	6	7	8	9	9	10
		50	8	9	9	10	11	11	11	8	9	10	11	11	11	12
		75	10	11	11	12	12	13	12	11	11	12	12	12	13	13
		95	13	13	13	14	14	14	14	14	13	14	14	14	14	14
2-back	*M*		4.10	5.02	5.23	6.29	6.96	7.26	8.16	3.96	4.53	5.74	6.52	7.44	7.93	9.11
	*SD*		3.64	3.67	3.81	3.78	4.08	3.91	3.99	3.59	3.49	3.76	3.50	3.68	3.77	3.75
	Percentile	5	–	–	–	–	–	–	–	–	–	–	–	–	–	2
		25	–	2	2	3	4	5	6	–	1	3	4	5	6	7
		50	4	5	5	6	7	8	9	3	5	6	7	8	8	10
		75	7	8	8	9	10	11	11	6	7	9	9	10	11	12
		95	11	11	12	12	13	13	13	11	10	12	12	13	13	14
3-back	*M*		2.30	2.87	3.26	4.23	4.97	5.04	6.37	2.07	2.45	3.41	4.44	5.38	6.05	6.80
	*SD*		3.41	3.81	3.91	4.15	4.27	4.12	4.27	3.46	3.46	3.98	4.24	4.28	4.36	4.11
	Percentile	5	–	–	–	–	–	–	–	–	–	–	–	–	–	–
		25	–	–	–	–	–	–	2	–	–	–	–	–	1	5
		50	–	–	–	4	6	6	7	–	–	–	5	6	7	8
		75	5	6	7	8	9	8	10	4	5	7	8	9	10	10
		95	9	10	11	11	11	11	12	10	10	10	11	12	12	13

## Discussion

The n-back task is a WM measure that is being used more frequently in research, clinical settings and in training studies. This research aimed to provide normative data collected from a large-scale cross-sectional study on a wide sample of children and adolescents aged 7–13 years. Participants were individually administered a computerized verbal n-back task with three levels of load: 1-back, 2-back, and 3-back. The results revealed clear effects in relation to the task’s level of difficulty as well as consistent age patterns in the different variables analyzed.

The results showed an overall improvement in performance with age, which, in general, translated into an increase in the percentage of hits and in the discrimination parameter *d*′ accompanied by a decrease in response times and in the percentage of false alarms. However, slightly different patterns were observed depending on the dependent variable and the task’s level of difficulty.

Gradual improvement in performance was observed in both hits and the discrimination index, *d*′, across the different age groups, which was more pronounced at 2-back than at 1-back. This implies that differences between both levels decrease progressively with age. Thus, while at age 7 the percentage of hits was about 66% in the easiest condition and 37% in the most difficult, at age 13 the difference between both levels decreased from 80% at 1-back to 65% at 2-back. This suggests that the age-related improvement was more pronounced at the 2-back level than at 1-back. The regression analyses showed a linear effect of age at the more complex levels (2-back and 3-back), while at 1-back a quadratic effect was also found. Other authors have reported that, at 1-back, a mature level is reached at 10–12 years, whereas at 2-back performance continues to improve even after adolescence (Brahmbhatt et al., 2010; Schleepen and Jonkman, 2010).

Interestingly, the age-related patterns found in hits were not observed in false alarms. In the case of hits at 1-back, there were only small differences between the older age groups. In contrast, the number of false alarms experienced a parallel decline with age at both n-back levels. This discrepancy implies that the mirror effect, a regularly occurring phenomenon in recognition memory tasks (Glanzer and Adams, 1985), does not occur across the different age levels included in this study. The mirror effect is observed on single “yes-no” recognition tasks when a factor that increases the hit rate decreases the false alarm rate or *vice versa*. For example, the advantage that word concreteness produces on recognition hit-rate is mirrored in the false alarms rate. In this study, in which age was the factor considered, it was found that the relationship between the ability to accurately recognize and reject items in the 1-back condition differed across the age groups.

The reduction of the load effect on hit-rate observed across the age groups was not found for response times. Thus, the difference between the times taken on both levels of difficulty was similar across all age groups. Other studies (e.g., Schleepen and Jonkman, 2010) have found a similar discrepancy between both variables. The disparity in the pattern between time and accuracy may indicate some independence in the development of factors related to storage and speed. In this regard, Bayliss et al. (2005) have suggested that both aspects can develop independently.

The results described above may have different but not mutually exclusive explanations. First, it is clear that both 1-back and 2-back involve different storage demands. The 1-back level requires one item to be stored in memory whereas 2-back requires two. In addition, both levels entail varying demands of information manipulation. Second, the 2-back condition requires greater executive control demands than the 1-back condition. It has been found that when executive control demands are not excessively high, children aged 11 may perform well (e.g., Best et al., 2009). However, when the conditions are more complex, clear improvements in performance in older age groups are detected, suggesting further executive development (e.g., Gathercole, 1999; Huizinga et al., 2006).

Also related to changes in executive control, Schleepen and Jonkman (2010) have reported that developmental differences are specifically due to an improvement in interference control. In support of this idea, these authors have found significant correlations between the difference in scores obtained on 1-back and 2-back levels and performance in a flanker task used as a measure of interference control. Thus, developmental changes in the ability to manage interference in WM could be a factor contributing to the age-related differences shown in this study.

An additional possible explanation for the age trends previously described is related to the ability to switch the focus of attention. At 1-back the last item presented, which is still held active in the focus of attention, should be compared with the new item; however, at 2-back the penultimate item must be retrieved into the focus of attention in order to compare it with the new one (e.g., McElree, 2001). Studies with older people have found age-related differences on n-back performance, which have been attributed to the increased cost of switching the focus of attention with age (e.g., Verhaeghen and Basak, 2005; Van Gerven et al., 2008). Recently, Lendínez et al. (2015) have shown that the ability to accurately retrieve information from outside the focus of attention changes across childhood and adolescence. Therefore, changes in the ability for focus switching could contribute to explain the age trends observed in this study.

Finally, the age patterns described may also reflect the differential contribution of familiarity and recognition mechanisms to the different levels of the n-back task. Responses in the 1-back level may be based on familiarity, whereas at 2-back and at higher levels, recollection is also necessary (e.g., Oberauer, 2005). It might be possible that changes related to the familiarity mechanism are more moderate than those of the recollection process. This pattern is also consistent with the results obtained with episodic tasks. Although changes in both processes have been observed since early childhood, familiarity seems to stabilize by mid-childhood, whereas recollection continues to improve during adolescence (for a review, see Ghetti and Lee, 2013).

To summarize, changes in executive control may be mediated by developmental improvements relating to different mechanisms such as interference resolution, focus switching, and even recollection. Note however, that we have not tested the different possible theoretical explanations of the observed age trends, since our aim was to provide normative data for different age groups. Hence, the investigation of the relative merits of these explanations may be a relevant objective for future studies. In addition, it would also be important to determine the specific relationship between performance at the different n-back levels and other complex WM measures for different age groups.

In this study, boys tended to respond more quickly than girls, although with less accuracy, committing more false alarms and obtaining fewer hits. These slight differences persisted across all ages. Even though the effect size of these differences was not large ($\eta_{p}^{2}$ between 0.001 and 0.018), the differences would suggest that boys exhibit greater impulsiveness (see also Vuontela et al., 2003). The findings are consistent with other studies reporting differences in favor of girls in some WM and STM tasks involving verbal processing (e.g., Lowe et al., 2003; Lynn and Irwing, 2008; however, for no differences see Nagel et al., 2007; Lejbak et al., 2011). Vuontela et al. (2003) have suggested that these gender differences could be due to differences in neurodevelopment. It has also been argued that boys and girls could use different strategies while performing the task (Speck et al., 2000).

The current study provided normative data on children and adolescents aged 7–13 years performing a computerized n-back task. The sample size allowed for accurate estimations of typical performance for the different age groups under study, and to determine at what age children were able to deal with the different levels of the task. In this regard, practically all children performed adequately at 1-back. However, a considerable number of 7-year-olds did not manage to complete the 2-back level or only achieved low levels of performance. Task difficulty increased notably at 3-back, given that most of the 7-year-olds children were not able to complete it. The results of the regression analyses indicated that the 2-back was the level that explained age-related performance best. Future studies should include older age groups in order to assess when asymptotic values are reached for the different levels of difficulty. It is possible that better performance at higher n-back levels might not be reached until after adolescence, a time when executive functioning seems to reach maturity (Best and Miller, 2010).

In addition to the clear age patterns found, high variability was observed in performance across the different ages. For example, at 1-back, there were participants from all age groups who obtained all possible scores. At 2-back the variability was reduced to some extent given that a number of younger children (i.e., aged 7) were unable to perform the task, and those who were, did not achieve the highest scores. Despite the difficulties of the task for the younger children, there was evidence of 7-year-olds performing the task at a level similar to that of many 13-year-old children. An analogous pattern emerged at 3-back. Although most children younger than 9 were unable to reach this level, nor did many of those who were older, there were participants across all ages who did manage to perform it.

These normative data could be used for the assessment of verbal WM and the updating function. Individual differences in WM capacity have important consequences for children’s ability to acquire knowledge and new skills (see Swanson and Alloway, 2012, for a review). The ability to update information has also been shown to have an important role in accounting for individual differences in different areas of academic attainment (e.g., Passolunghi and Pazzaglia, 2004; Swanson and Beebe-Frankenberger, 2004; St Clair-Thompson and Gathercole, 2006; Van der Sluis et al., 2007; Carretti et al., 2009; Cornoldi et al., 2012; Lechuga et al., 2015; Pelegrina et al., 2015). Low scores for a specified age on the n-back task could indicate that this system, involved in numerous complex cognitive abilities, is functioning poorly. Therefore, these normative data could contribute to detect children at risk of academic difficulties and thus support early intervention programs and strategies.

These normative data may also be valuable in the context of WM training programs. There is some evidence that WM capacity can be increased by training, at least in the short-term (e.g., Jaeggi et al., 2014). The effects of WM training may also transfer to academic attainment (for a review, see Titz and Karbach, 2014). The n-back is a task frequently used to train WM and to assess WM capacity in both children and adults (see Melby-Lervag and Hulme, 2012). The task presented in this study could be used to obtain baseline scores and to determine the possible changes after training. Baseline scores and possible variations after the training could be readily interpreted in relation to the corresponding age group.

There are some limitations in the present study that suggest ideas for future research. First, it should be noted that the variables considered in the regression analyses explained a small portion of the variance. This suggests that additional factors are influencing performance on this task as well as individual differences. The n-back task requires multiple executive control processes and these could be a source of performance variability. It has been shown that executive functions component processes may develop at different rates (Huizinga et al., 2006; Best et al., 2009; Xu et al., 2013). Thus, it might be possible that different processes involved in the task contribute to the observed age patterns. Further studies should analyze the specific cognitive processes that are involved in this task.

Furthermore, although the cross-sectional design may be useful to identify age differences, only a longitudinal design permits the capture of intra-individual changes over time. Thus, for instance, it might be relevant to analyze different developmental patterns (e.g., early- or late-maturing children). In addition, a longitudinal design would be informative not only in regards to the individual differences in performance shown in this study, but also in regards to their possible changes over time. Moreover, this design would also permit determining the relationship over time between some cognitive processes and performance on the n-back task.

Finally, it should also be relevant to compare the results obtained with different n-back tasks. The n-back task we administered followed a procedure analogous to that used in other studies. However, given that there is no standard procedure for this task, it should be appropriate to investigate the extent to which these results can be generalized to other versions.

To conclude, the results indicate age-related changes that might be related to different cognitive processes involved in updating information in WM. The normative data obtained may be useful in interpreting the results of other studies in which the n-back task has been applied under similar conditions. Furthermore, given the type of stimuli used, this information might be valuable even when the task is applied in other languages. The normative data collected in this study may be relevant on a practical level, given that they provide information about performance on a WM task that taps executive processes that are central to cognitive development and learning.

### Conflict of Interest Statement

The authors declare that the research was conducted in the absence of any commercial or financial relationships that could be construed as a potential conflict of interest.

## Footnotes

1 A parallel set of analyses including all the levels of n-back load was run. The main effects and interactions were roughly equivalent to those presented when considering only the 1-back and 2-back levels. Importantly, the age linear trend for 3-back was significant on hits: *F*(1,2134) = 103.63, *p* < 0.001, $\eta_{p}^{2}$ = 0.046; false alarms: *F*(1,2134) = 90.58, *p* < 0.001, $\eta_{p}^{2}$ = 0.041; d-prime: *F*(1,2134) = 180.96, *p* < 0.001, $\eta_{p}^{2}$ = 0.078; and response times: *F*(1,2086) = 78.49, *p* < 0.001, $\eta_{p}^{2}$ = 0.036.
